# Molecular mechanisms underlying exercise-enhanced autophagy in improving neuroplasticity in Alzheimer’s disease

**DOI:** 10.3389/fnagi.2026.1780247

**Published:** 2026-03-12

**Authors:** Qian Li, Renqing Zhao, Xin Tian, Haocheng Xu

**Affiliations:** 1 Department of Sports Human Sciences, College of Physical Education, Yangzhou University, Yangzhou, Jiangsu, China; 2 Department of Neuroscience and Physiology, SUNY Upstate Medical University, Syracuse, NY, United States

**Keywords:** Alzheimer’s disease, autophagy, cognitive functions, exercise, neuroplasticity

## Abstract

Alzheimer’s disease (AD), the most prevalent form of dementia, is characterized by progressive memory impairment and cognitive dysfunction. The neuropathological hallmarks of this neurodegenerative disorder encompass two principal pathological features: extracellular deposition of amyloid-*β* (Aβ) plaques due to abnormal protein aggregation, and intracellular accumulation of neurofibrillary tangles (NFTs) caused by hyperphosphorylation of tau proteins (p-Tau). These pathological changes induce synaptic loss and neuronal apoptosis, which leads to impaired neuroplasticity and progressive deterioration of cognitive function. Autophagy, a critical mechanism in the central nervous system (CNS) responsible for clearing misfolded protein aggregates and damaged organelles, plays a pivotal role in maintaining neuronal homeostasis and synaptic plasticity. However, AD is associated with autophagy impairment, resulting in the accumulation of toxic protein aggregates and damaged organelles. These pathological changes disrupt protein homeostasis, thereby exacerbating neurodegenerative processes. Currently, AD therapeutic strategies remain limited. Emerging evidence indicates that exercise intervention mitigates cognitive decline and enhances synaptic plasticity, potentially through reducing Aβ deposition and pathological phosphorylation of tau proteins. However, the precise mechanisms through which these interventions act remain to be fully elucidated. Recent studies have shown that exercise can promote autophagosome formation, fusion, and lysosomal hydrolytic function, thereby ameliorating the pathological progression of AD. Despite these promising findings, the precise molecular targets and underlying signaling mechanisms through which exercise modulates autophagy in AD remain to be fully elucidated. The purpose of this study is to establish innovative therapeutic targets while identifying mechanistically actionable pharmacological targets to advance therapeutic development against AD pathogenesis.

## 1 Introduction

AD represents the primary etiology of dementia, responsible for 60–80% of all diagnoses (Livingston et al., 2024; Beura et al., 2023). This neurodegenerative condition involves unremitting cognitive deterioration driven by irreversible neuronal injury. Driven by global demographic shifts toward an older population, the incidence of AD is climbing steeply. Current epidemiological models forecast a near-tripling of cases from 55 million in 2020 to 152 million by 2050, imposing an annual socioeconomic burden surpassing US$2.8 trillion (Cumming and Brodtmann, 2010). Consequently, AD has emerged as a preeminent public health priority, underscoring the critical need for innovative clinical intervention strategies. In traditional therapeutic strategies for AD, drug interventions primarily rely on cholinesterase inhibitors (such as donepezil) and N-methyl-D-aspartate (NMDA) receptor antagonists (such as memantine) to alleviate symptoms (Beura et al., 2023; Sharma, 2019). However, the therapeutic landscape has undergone a significant shift recently. Regulatory authorities in several countries, including the United States, Japan, and China, have successively approved monoclonal antibodies targeting Aβ Key examples of these treatments include lecanemab and donanemab. This approach focuses on the targeted clearance of amyloid plaques within the brain. It represents a significant breakthrough in disease-modifying treatments for AD. However, its long-term clinical benefits and safety profiles still require more evidence-based support. A major concern is the risk of amyloid-related imaging abnormalities (ARIA). This includes ARIA-oedema/effusion (ARIA-E) and ARIA-haemosiderosis/microhaemorrhages (ARIA-H). Consequently, rigorous clinical monitoring and extended follow-up studies are essential (van Dyck et al., 2023; Sims et al., 2023; Hampel et al., 2023). Consequently, there is an urgent demand for the development of alternative, effective therapeutic strategies. The etiology of AD is driven by a dual neuropathology involving the aggregation of amyloid-*β* and the aberrant phosphorylation of tau. These pathological events fundamentally compromise neuronal network integrity, driving synaptic dysfunction and the loss of neuroplasticity that underlie the clinical progression of cognitive decline (Dalal et al., 2024). Given these pharmacological limitations, physical exercise has emerged as a promising therapeutic strategy, capitalizing on its ability to modulate multiple pathological targets simultaneously. Evidence indicates that regular exercise effectively counters AD-associated hippocampal atrophy and cognitive decline. Mechanistically, this intervention promotes the secretion of brain-derived neurotrophic factor (BDNF), augments cerebral blood flow, and restores neuronal metabolic homeostasis (Stern et al., 2020). Emerging evidence suggests that physical exercise facilitates the restoration of neural plasticity by upregulating autophagic flux. By enhancing lysosomal clearance efficiency, this process effectively mitigates oxidative damage and neuroinflammation while restoring mitochondrial homeostasis, thereby countering the pathological environment of AD (Wang et al., 2022; Zhang W. et al., 2021; Zhang X. X. et al., 2021; Zhao et al., 2023; Jian et al., 2022). However, the precise molecular signaling cascades and the intricate crosstalk governing these neuroprotective effects have yet to be fully delineated. This review systematically dissects the regulatory networks governing exercise interventions across four pivotal dimensions: the modulation of neuroinflammation, the restoration of redox homeostasis, mitochondrial quality control, and the enhancement of lysosomal function. By integrating these perspectives, we elucidate the molecular mechanisms through which exercise orchestrates neural plasticity in AD. Ultimately, this work establishes a theoretical framework and a translational roadmap for developing novel preventative and therapeutic paradigms targeted at the physical exercise-autophagy axis.

## 2 Autophagy dysfunction and neuroplasticity in AD

### 2.1 Autophagy dysfunction in AD

AD is biologically characterized by the deposition of Aβ protein both extracellularly and intracellularly, as well as the excessive phosphorylation of tau protein within cells. Although extracellular plaques remain the hallmark pathological feature, the accumulation of Aβ within neurons is increasingly recognized as a key early driver of neurotoxicity and the collapse of neural network architecture (Knopman et al., 2021; Serrano-Pozo et al., 2011; Dalal et al., 2024). Autophagy operates as a fundamental cellular renewal and defense mechanism, dynamically sequestering misfolded proteins and dysfunctional organelles within double-membrane autophagosomes. This sequestration facilitates the catabolism of aberrant substrates by lysosomal hydrolases, thereby upholding neuronal proteostasis and conferring robust neuroprotection (Sose et al., 2023). Biologically, autophagy acts as a multi-step cascade encompassing six distinct stages: initiation, nucleation, elongation, maturation, fusion, and degradation. The dynamic efficiency of this entire cycle is termed “autophagic flux.” An arrest at any specific stage compromises this flux, precipitating the buildup of toxic protein aggregates which ultimately drives neuronal degeneration (Li et al., 2021). The initiation of autophagy depends on the activation of the Unc-51-like autophagy-activating kinase 1 (ULK1) complex, whose activity is regulated by the nutrient-sensing signal mechanistic target of rapamycin complex 1 (mTORC1) and the energy-sensing signal AMP-activated protein kinase (AMPK). Under nutrient-sufficient conditions, mTORC1 phosphorylates ULK1 at the Ser757 site, disrupting the interaction between ULK1 and AMPK. This inhibits ULK1 kinase activity and restricts autophagy. Conversely, during energy stress or exercise-induced states, AMPK phosphorylates ULK1 at Ser317 and Ser777 sites, enhancing its kinase function and thereby promoting autophagy initiation (Kim et al., 2011; Siva Sankar et al., 2022). This regulatory mechanism enables dynamic modulation of autophagy in response to cellular energy status and external stimuli (Mary et al., 2025). The nucleation of the isolation membrane is critically orchestrated by Bcl-2-interacting protein 1 (Beclin 1). According to work by Pickford et al. (2008), a deficiency in this protein is a hallmark of AD pathology. In *APPswe/PS1dE9* mouse models, the genetic reduction of Beclin 1 expression was found to disrupt vesicle nucleation, leading to an increased burden of Aβ plaques and a concomitant loss of synaptic proteins. The pathological accumulation of p-Tau disrupts neuronal microtubule integrity, thereby creating a physical blockade to organelle transport. As a result, massive quantities of autophagosomes are trapped in distal neurites and synaptic terminals. This trafficking defect constitutes a primary mechanism underlying the stagnation of autophagic flux (Tammineni and Cai, 2017). During the elongation phase, autophagy-related gene ATG4 (ATG4) orchestrates the processing of microtubule-associated protein 1 light chain 3 (LC3). Specifically, ATG4 cleaves the LC3 precursor to generate cytosolic LC3-I. This soluble form is subsequently conjugated to phosphatidylethanolamine (PE) to yield lipidated LC3-II. Crucially, LC3-II anchors to the autophagosomes double membrane and remains associated until fusion with the lysosome. Simultaneously, sequestosome 1 (SQSTM1/p62) functions as a key cargo receptor that identifies ubiquitinated aggregates. Upon binding, p62 targets these substrates to the nascent autophagosome by interacting with lipidated LC3, leading to their lysosomal degradation. Thus, measuring the LC3-II/I conversion ratio provides an index of autophagic induction intensity. Conversely, p62 levels reflect the degradative capacity of the system, where its accumulation is indicative of blocked autophagic flux (Jiang and Mizushima, 2015). Under normal circumstances, autophagic vacuoles (AV) are present in limited quantities in a healthy brain. However, Zhou et al. (2024) observed a marked co-elevation of LC3-II and p62 in the hippocampal tissue of AD mice. This concurrent accumulation signifies that while autophagosomes are formed, they are not efficiently cleared, indicating a state of blocked autophagic flux. Mechanistically, it is hypothesized that Aβ oligomers may aberrantly interact with LC3 or the autophagosomes membrane, thereby physically impeding fusion with lysosomes. This disruption results in the pathological stagnation of autophagosomes and the failure of cargo degradation (Lee et al., 2022). The fusion and degradation phases of autophagy are critically dependent on lysosomal integrity. The functionality of these organelles is tightly controlled by Transcription Factor EB (TFEB). Currently evidence suggests that the lysosomal failure observed in AD is inextricably linked to aberrant TFEB activity. Relevant studies indicate that Aβ deposition and oxidative stress synergistically impair the nuclear translocation of TFEB. This blockade suppresses lysosomal biogenesis, thereby compromising the degradative capacity of the autophagy-lysosome pathway (Zhang and Zhao, 2015).

### 2.2 The interplay between autophagy dysfunction and neuroplasticity in AD

Neuronal plasticity, the neurobiological substrate for cognitive function, facilitates the adaptive remodeling of neural networks in response to behavioral changes. This broad concept encompasses two fundamental pillars: synaptic plasticity and adult hippocampal neurogenesis (AHN). Specifically, AHN is defined as the process wherein neural stem cells (NSCs) proliferate and differentiate to generate functional new neurons (Chang et al., 2024). However, in the early stages of AD, this adaptive machinery is severely compromised. The pathological accumulation of Aβ and p-Tau precipitates a marked impairment in these plastic processes, thereby driving cognitive decline (Stern et al., 2020). In the neuropathological trajectory of AD, the hippocampus represents one of the earliest and most severely compromised regions. Within its dentate gyrus, the subgranular zone (SGZ) serves as the structural foundation for neurogenesis. This region concentrates the regulatory machinery required for the proliferation and differentiation of NSCs (Boese et al., 2018). Studies indicate that as AD progresses, the buildup of misfolded protein aggregates impairs the functionality of NSCs, specifically stifling their proliferation and differentiation. This results in a marked suppression of hippocampal neurogenesis (Cosacak et al., 2020). ATG5 is a core mediator of autophagy. It is specifically required for autophagosome expansion and LC3 lipidation (Klionsky et al., 2021; Ge et al., 2014). Evidence suggests that ATG5-dependent autophagy primarily clears Aβ peptides and their amyloidogenic C-terminal fragments (CTFs), rather than degrading the physiologically functional APP precursor protein itself (Tian et al., 2011). Dysfunction in ATG5-mediated autophagy leads to the intracellular accumulation of these toxic proteins. This accumulation subsequently promotes extracellular plaque formation and accelerates neurodegeneration (Hein et al., 2025). By clearing these abnormal aggregates, autophagy maintains neuronal proteostasis and supports synaptic plasticity (Hwang et al., 2019). Synaptic plasticity serves as the neurobiological foundation for cognition, learning, and memory, manifesting primarily as long-term potentiation (LTP) and long-term depression (LTD) (Mango et al., 2019). At the molecular level, these functional dynamics are underpinned by structural integrity. Consequently, postsynaptic density protein 95 (PSD-95), a key postsynaptic scaffolding protein, and synaptophysin (SYN), a major presynaptic vesicle protein, are widely utilized as canonical markers to evaluate synaptic stability and density (Li et al., 2020). Observations in *APP/PS1* mice further confirm that impaired synaptic plasticity, manifested as reduced LTP, is underpinned by deficits in synaptic density and morphology. Furthermore, the inactivation of autophagy has been identified as a key mechanism contributing to this process. The resulting accumulation of aberrant proteins in the synaptic zone disrupts neural network integrity, leading to cognitive impairment (Grosso Jasutkar et al., 2024). Furthermore, Aβ-induced dysfunction of dynamin-1 impairs synaptic vesicle endocytosis and recycling, leading to synaptic depletion (Rajmohan and Reddy, 2017). Simultaneously, Aβ oligomers block glutamate reuptake, causing extracellular accumulation. This drives NMDA receptor-mediated excitotoxicity, which directly fuels synaptic dysfunction and the loss of neuronal plasticity (Talantova et al., 2013). Taken together, these findings suggest that autophagic failure in AD disrupts the clearance of proteopathic aggregates, ultimately exacerbating the damage to neuronal plasticity.

Preserving neuroplasticity is essential for resisting neurodegenerative pathology. By modulating NSC proliferation and maintaining synaptic proteostasis, autophagy protects synaptic structure and function, thereby underpinning the brain’s plastic capacity (Fleming and Rubinsztein, 2020). Collectively, these findings suggest that re-establishing autophagic balance is a viable therapeutic strategy to counteract neuroplasticity loss in AD. While the potential of autophagy activation is clear, the specific molecular mechanisms driving this protective effect warrant further investigation.

## 3 Regulatory mechanisms underlying autophagy dysfunction in AD

Given the established link between autophagy dysfunction and neuroplasticity deficits in AD, it is imperative to dissect the upstream drivers of this failure (Mango et al., 2019). The etiology of AD is highly multifaceted, suggesting that autophagy is not regulated in isolation but through a complex, multi-target network. Recent evidence has elucidated this interconnected regulation. Therefore, this section will systematically examine the mechanisms underlying autophagy dysfunction from four critical dimensions: neuroinflammation, oxidative stress, mitochondrial dysfunction, and lysosomal impairment.

### 3.1 Neuroinflammation and autophagy dysfunction

Neuroinflammation encompasses the complex inflammatory immune response within the CNS elicited by diverse endogenous or exogenous stimuli, including pathogens and misfolded protein aggregates. It is widely recognized as a prominent pathological hallmark of AD (Heidari et al., 2022). Microglia, the resident macrophages of the CNS, utilize autophagy to maintain homeostasis by clearing pathological debris. Under conditions of chronic inflammation, however, this autophagic flux is significantly impaired. This impairment prevents the metabolic switch required for microglial recovery, locking them in a neurotoxic M1 state. The resulting impaired protein degradation, coupled with the sustained release of pro-inflammatory cytokines such as Tumor necrosis factor-*α* (TNF-α) and interleukin-1β (IL-1β), directly triggers a cascade of events ultimately leading to neuronal death (Zhao, 2024). Specifically, the excessive activation of microglia and the subsequent release of IL-1β suppress the expression of BDNF, thereby impairing neuronal plasticity (Biswas, 2023). Evidence indicates that chronically elevated IL-1β promotes neuronal death by triggering caspase-3-mediated apoptotic pathways and inducing mitochondrial membrane potential collapse (Calsolaro and Edison, 2016). Furthermore, the NLRP3 inflammasome (NOD-like receptor family pyrin domain containing 3) constitutes a pivotal component of the innate immune system (Wang S. et al., 2024; Wang T. et al., 2024). Its assembly facilitates the activation of caspase-1, which subsequently catalyzes the proteolytic cleavage and maturation of key pro-inflammatory cytokines, specifically IL-1β and interleukin-18 (IL-18) (Liang et al., 2022). Mechanistically, autophagy acts as a brake on neuroinflammation. It drives microglial polarization toward the reparative M2 phenotype, reducing the release of cytotoxic cytokines. Crucially, autophagy targets Aβ for lysosomal degradation. By removing this upstream trigger, it effectively abrogates Aβ-induced NLRP3 inflammasome assembly and activation (Chen et al., 2024). Crucially, the resulting inhibition of IL-1β secretion suppresses the activity of the NF-κB pathway. This molecular blockade not only attenuates cerebral inflammatory responses and Tau accumulation but also induces the expression of BDNF. Collectively, these changes curtail the release of inflammatory mediators, thereby exerting a potent neuroprotective effect (Kitazawa et al., 2011). Furthermore, TNF-*α* acts as a critical modulator of APP metabolism. It potentiates the activity of both Beta-site Amyloid Precursor Protein Cleaving Enzyme 1(BACE1) and *γ*-secretase, facilitating the cleavage of APP into neurotoxic Aβ40 and Aβ42. The resulting deposition of fibrillar plaques reinforces a vicious cycle of aberrant APP processing, ultimately fueling the rapid progression of AD (Kumar and Bansal, 2022). Consequently, excess TNF-α may impair the clearance of neurotoxic aggregates by inhibiting autophagy, thereby disrupting synaptic plasticity and LTP, which are associated with AD-related cognitive decline.

### 3.2 Oxidative stress and autophagy dysfunction

Oxidative stress is recognized as a pivotal pathogenic mechanism underlying AD. Mutations in AD-associated genes can significantly elevate cerebral oxidative stress levels, thereby accelerating the formation of Aβ plaques and inducing neuronal oxidative damage (Dhapola et al., 2024). Notably, Aβ deposition serves not only as a pathological hallmark of the disease but also as a potent catalyst that further promotes the generation of oxidative stress. As the central hub for reactive oxygen species (ROS) production, mitochondria suffer from profound structural and functional deficits in the AD brain. Mechanistically, Aβ deposits on the mitochondrial membrane and suppresses cytochrome c oxidase activity. This blockade disrupts the integrity of the electron transport chain, thereby triggering an excessive surge in ROS levels (Wang et al., 2020). In essence, oxidative stress arises when the production of ROS exceeds the neutralizing capability of antioxidant enzymes, particularly superoxide dismutase (SOD), heme oxygenase-1 (HO-1), and catalase (CAT) (Bhatia and Sharma, 2021). Given the brain’s exceptionally high oxygen demand, it is uniquely vulnerable to oxidative stress. Excess ROS activates the GSK-3β pathway, leading to p-Tau. This pathology disrupts axonal mitochondrial transport, causing the stasis of damaged mitochondria. Consequently, local ROS production is amplified, fueling a vicious cycle that compromises axonal integrity (Liu et al., 2015). Concurrently, NSCs exhibit distinct responses to oxidative stress levels. Under mild stress, they mobilize antioxidant enzymes like SOD to counteract damage. In contrast, sustained severe stress overwhelms these defenses, impairing NSC proliferation and differentiation and thereby halting endogenous neurogenesis (Chohan, 2020). Importantly, autophagy is capable of selectively eliminating oxidized substrates while maintaining essential redox signal transduction, thus preventing ROS overload (Zhang W. et al., 2021; Zhang X. X. et al., 2021). Central to this regulation is p62, which functions as a signaling scaffold. Through its PB1 domain, p62 orchestrates oxidative stress response pathways, driving the expression of antioxidant genes and effectively restoring intracellular redox homeostasis (Monzel et al., 2023). In summary, autophagy acts as a protective buffer against ROS, essential for maintaining redox stability. Thus, targeting this pathway offers a potential method for ameliorating neuroplasticity deficits.

### 3.3 Mitochondrial dysfunction and mitophagy impairment

Mitochondria act as the bioenergetic command centers of the neuron. Beyond their canonical role in Adenosine Triphosphate (ATP) synthesis, they are integral to calcium buffering, redox regulation, and the maintenance of synaptic plasticity, ultimately determining neuronal viability (Cadete et al., 2019). Notably, during synaptogenesis and neural signal transmission, mitochondria are specifically recruited to presynaptic terminals. The ATP generated by these organelles not only fuels synaptic vesicle trafficking but also regulates neurotransmitter release, thereby ensuring the integrity and stability of synaptic function (Mary et al., 2023). Furthermore, during early neuronal differentiation, mitochondria regulate axonal outgrowth and elongation by orchestrating local calcium (Ca^2+^) dynamics. Beyond development, mitochondrial ATP synthesis acts as a crucial determinant for the modulation and maintenance of synaptic plasticity. During AD pathology, the deposition of Aβ aggregates and p-Tau disrupts mitochondrial homeostasis. These factors synergistically blockade the electron transport chain (ETC) and oxidative phosphorylation, leading to an energy crisis and an aberrant surge in ROS. This mitochondrial stress injury not only undermines synaptic function but also precipitates neuronal death, thereby propelling the progression of neurodegenerative lesion (Wang et al., 2020; Rehman et al., 2023). Relevant studies have demonstrated that mitophagy levels in the brain tissue of AD patients are reduced to approximately 50% of those observed in healthy controls. This significant decline provides direct evidence of compromised mitochondrial clearance mechanisms (Fang et al., 2019). As a selective autophagic process dedicated to the clearance of damaged mitochondria, mitophagy is pivotal for maintaining mitochondrial quality control and energy homeostasis. Consequently, it serves as a fundamental mechanism regulating neuronal function and plasticity (Wang et al., 2023). The PTEN-induced kinase 1 (PINK1)/Parkin RBR E3 ubiquitin-protein ligase (Parkin) pathway is the definitive mechanism governing mitophagy. Following mitochondrial depolarization, PINK1 accumulates on the outer membrane, recruiting cytosolic Parkin to orchestrate the ubiquitination of Outer Mitochondrial Membrane (OMM) proteins, thereby creating binding sites for receptors like p62. This facilitates the sequestration of mitochondria into autophagosomes for lysosomal degradation (Cadete et al., 2019). Evidently, this pathway serves as a cornerstone of cellular homeostasis. By eliminating dysfunctional, ROS-producing mitochondria, PINK1/Parkin-mediated mitophagy preserves bioenergetic efficiency and safeguards neuronal stability.

### 3.4 Lysosomal dysfunction and blockade of autophagic flux

As the central hub for autophagic degradation, lysosomes are indispensable for cellular quality control and the removal of harmful aggregates. Serving as the culmination of the autophagic pathway, the effective fusion with lysosomes and the degradation of substrates are absolute prerequisites for the successful execution of autophagy (Li et al., 2021). Accumulating evidence indicates that lysosomal dysfunction is a primary driver of reduced autophagic efficiency in AD. Primarily, this involves Lysosomal-Associated Membrane Proteins (LAMPs), particularly LAMP1 and LAMP2, which are essential for mediating autophagosome-lysosome fusion and maintaining lysosomal homeostasis. In AD pathology, aberrant APP metabolism and functional defects in Presenilin-1 (PS1) lead to the dysregulated expression of LAMP proteins. This disruption impedes the fusion of autophagosomes with lysosomes, resulting in the inefficient degradation of cellular debris and toxic aggregates. Consequently, the trafficking of autophagy proteins is hindered, triggering a pathological accumulation of undigested substrates (Lo and Zeng, 2023). Secondly, the degradative efficacy of lysosomes hinges on a suite of luminal hydrolases. Cathepsin D (CTSD) is the predominant aspartic protease within the lysosomal lumen. Its primary function is to drive the proteolytic degradation of APP, Aβ and phosphorylated Tau. Notably, the accumulation Aβ42 can inhibit CTSD’s maturation and activity. This enzymatic inhibition subsequently triggers a pathological cascade, leading to progressive Tau accumulation and neurodegeneration (Terron et al., 2024). Alongside CTSD, Cathepsin B (CTSB) functions as a critical cysteine protease and a marker of lysosomal maturation. In AD pathology, the activity of such proteases is significantly compromised. This enzymatic deficit reduces the clearance of neurotoxic Aβ and exacerbates its aggregation. Specifically, impaired CTSB activity disrupts the turnover of synaptic proteins, such as PSD-95, thereby destabilizing synaptic structure (Lee et al., 2022). Supporting this connection, research shows that calcium channel blocker treatment can partially restore Cathepsin activity, leading to the amelioration of synaptic deficits and the recovery of LTP. These findings provide empirical evidence of a direct causal relationship between lysosomal failure and the loss of synaptic plasticity (Mustaly-Kalimi et al., 2022). Finally, the maintenance of an acidic luminal environment (pH ~ 4.5–5.0) is paramount for sustaining hydrolase activity and ensuring membrane fusion competency. This acidification is driven by the vacuolar type H^+^-ATPase (v-ATPase), a multimeric proton pump complex essential for lysosomal homeostasis (Huang et al., 2019), However, in AD, mutations in PS1 and APP have been shown to impair v-ATPase maturation and function. The resulting defective acidification compromises the activity of degradative enzymes, thereby diminishing the clearance capacity for neurotoxic proteins such as Aβ (Lee et al., 2022). Collectively, the impairment of membrane fusion capacity, hydrolytic degradation function, and luminal pH homeostasis defines lysosomal dysfunction in AD. These multifaceted defects constitute critical bottlenecks that arrest autophagic flux, thereby exacerbating the accumulation of neurotoxic burdens and compromising neuronal plasticity.

## 4 Physical exercise ameliorates neuroplasticity in AD

Currently, non-pharmacological interventions for AD predominantly fall into three core categories: cognitive training, lifestyle modifications (encompassing sleep hygiene and nutritional interventions), and music therapy (Livingston et al., 2024). In contrast, long-term regular exercise confers pleiotropic benefits. It not only stimulates neuronal proliferation and differentiation, promotes the secretion of neurotrophic factors, and enhances neural plasticity, but also improves cardiopulmonary function and elevates overall quality of life (Zimmerman et al., 2024). According to research findings, approximately 35% of AD cases are attributable to nine modifiable risk factors, among which physical inactivity emerges as a primary contributor (Zhang W. et al., 2021; Zhang X. X. et al., 2021). Specifically, aerobic activities like brisk walking and dancing promote the maintenance of white matter microstructure in regions critical for cognitive processing, signaling a robust potential for neural plasticity. For instance, a study on holistic physic-cognitive rehabilitation (HPCR) revealed that despite a parallel decline in cognitive scores (Mini-Mental State Examination-MMSE) in both groups, the intervention crucially sustained Activities of Daily Living (ADL) in AD and MCI patients, preventing the significant functional loss observed in untreated controls (Osawa et al., 2025). Zhang et al. (2022) systematically reviewed and meta-analyzed 16 randomized controlled trials (RCTs), involving 503 participants in the exercise intervention group (mean age 69.2–84 years) and 406 in the control group (mean age 68.9–84 years). Analysis results indicate that aerobic exercise intervention can improve cognitive function in AD patients, with the most significant effects observed in a regimen involving single 30-min sessions totaling less than 150 min per week and no more than three sessions per week. Furthermore, patients with lower baseline cognitive levels tended to experience greater cognitive improvement through exercise intervention (Zhang et al., 2022). Aerobic exercise promotes AHN and significantly upregulates the expression levels of BDNF, IL-6, fibronectin domain-containing protein 5 (FNDC5), and multiple synapse-related markers. In an AD model (5 × FAD transgenic mice), these molecular changes collectively mediate cognitive improvement and exhibit pronounced anti-inflammatory neuroprotective effects (Choi et al., 2018). Specifically, following aerobic exercise intervention in *APP/PS1* transgenic mice, results from the Morris Water Maze (MWM)—the gold-standard assay for spatial cognition—revealed significant behavioral improvements (Wang S. et al., 2024; Wang T. et al., 2024). Exercised mice exhibited a marked reduction in escape latency and an increase in platform crossings. These findings indicate that spatial learning and memory capabilities were significantly ameliorated, outcomes that were concomitant with elevated synaptic density and the upregulation of PSD-95, a pivotal postsynaptic scaffolding protein (Yang et al., 2023).

Multiple human and animal studies have demonstrated that exercise exerts positive regulatory effects on brain health (Fragala et al., 2019). Current research in this field has primarily focused on aerobic exercise, while systematic investigations into resistance training remain relatively scarce. Resistance training is integral to comprehensive exercise plans for older adults. It requires skeletal muscles to contract against external resistance. This process effectively mitigates age-related declines in neuromuscular function and daily physical capacity. Additionally, this type of training significantly enhances muscle strength, promotes muscle mass growth, and comprehensively optimizes various muscle-related motor function performances (Borde et al., 2015; Rahmati et al., 2023). Findings from another clinical trial demonstrated that a 12-week resistance training regimen in AD patients elicited a marked upregulation in BDNF and Insulin-like Growth Factor-1(IGF1) expression. Concurrently, the study reported an approximate 30% elevation in markers of hippocampal neurogenesis (Azevedo et al., 2023; Talbot et al., 2012). Beyond clinical evidence, this therapeutic potential is further substantiated by preclinical studies. Research has revealed that resistance training increases the expression of synaptophysin-1 in synaptic vesicles within the frontal cortex. It also reduces *β*-amyloid deposition, tau hyperphosphorylation, and total tau burden in the brains of 3xTg mice (Sepúlveda-Lara et al., 2024; Liu et al., 2020). A study by Pena et al. comparing two exercise modalities found that aerobic exercise increased hippocampal IGF-1 concentrations in 3xTg mice and significantly prolonged peak latency in the rotarod test, thereby improving motor coordination and endurance. In contrast, resistance training elevated hippocampal IGF-1 levels in mice, markedly enhanced grip strength, and reduced hippocampal β-amyloid levels by approximately 30% (Pena et al., 2020).

To understand how exercise restores neural plasticity, we must examine the molecular landscape. Growing evidence suggests that upregulating autophagy provides a powerful neuroprotective shield (Tang et al., 2024). Notably, physical exercise acts as a potent stimulus for hippocampal autophagy and neurogenesis. In particular, aerobic training activates the autophagic machinery, evidenced by the increased expression of core components such as LC3, Beclin1, ATG7, and p62, alongside the upregulation of Neuregulin-1 (NRG1) (Wang et al., 2025; Jang, 2020). Corroborating these findings, Wu J. et al. (2024) and Wu J. J. et al. (2024) reported similar observations, demonstrating that physical exercise enhances neuronal autophagic activity in the hippocampus of AD mice. Structurally, this functional upregulation was accompanied by increased dendritic arborization and spine density in the CA1 region. These adaptive changes suggest that exercise bolsters the brain’s endogenous repair systems, thereby improving synaptic plasticity (Wu J. et al., 2024; Wu J. J. et al., 2024). Collectively, the studies substantiate the potential clinical utility of physical exercise as a non-pharmacological intervention for ameliorating neurodegenerative conditions such as AD. In the following sections, we will specifically elucidate the underlying molecular mechanisms.

## 5 Key signaling pathways linking exercise to autophagy-mediated neuroplasticity

### 5.1 Exercise modulates the PI3K/AKT/mTOR axis: bridging autophagy, inflammation, and neuroplasticity

Phosphatidylinositol 3-kinase (PI3K) constitutes a family of membrane-associated lipid kinases that catalyze the production of lipid second messengers, thereby orchestrating fundamental cellular processes including survival, autophagy, and energy metabolism. Protein kinase B (AKT), a critical serine/threonine kinase, is activated downstream of PI3K signaling. Once active, AKT modulates a diverse array of effectors, including the mammalian target of rapamycin (mTOR), GSK-3*β*, and Cyclic Adenosine Monophosphate (cAMP) response element–binding protein (CREB). Importantly, these mechanisms have been extensively characterized in AD rodent models. Notably, mTOR serves as a central negative regulator of autophagy and a primary target of the PI3K/AKT axis. Through this cascade, the pathway exerts pivotal control over synaptic plasticity-related protein expression and neuroinflammatory responses, highlighting its indispensable role in neuronal homeostasis (Rai et al., 2019; Ebrahim et al., 2024). This signaling axis plays an indispensable role in maintaining neuronal homeostasis. Specifically, aerobic exercise-induced AMPK activation represses mTOR while directly activating ULK1. This coordinated signaling cascade effectively initiates the autophagic process, bolstering the cell’s ability to cope with metabolic stress. Furthermore, enzyme-linked immunosorbent assay (ELISA) indicated that this exercise intervention also markedly decreased insoluble Aβ levels in the hippocampus and cortex of Alzheimer’s disease model mice (Wang and Jia, 2023; Wu J. et al., 2024; Wu J. J. et al., 2024). Conversely, aerobic treadmill exercise can also engage the PI3K/Akt signaling cascade, resulting in the inhibitory phosphorylation of GSK-3β and the indirect modulation of mTOR activity. Through this mechanism, exercise bolsters autophagic flux, thereby facilitating the degradation and clearance of Aβ and p-Tau. Ultimately, these molecular events contribute to the amelioration of cognitive impairment associated with AD (Pan et al., 2024; Peng et al., 2022) For instance, a 12-week treadmill running regimen has been shown to suppress the release of pro-inflammatory cytokines. Mechanistically, this anti-inflammatory effect was mediated by inhibiting the pathological activation of mTOR downstream of the PI3K/Akt signaling axis (Kang and Cho, 2015). Specifically, while microglia utilize the Toll-like receptor 4(TLR4)/PI3K/Akt/NF-κB axis to drive pro-inflammatory cytokine release (TNF-*α*, IL1-β), aerobic exercise intervention effectively suppresses this signaling cascade in AD rodent models. This suppression of neuroinflammation is pivotal for strengthening memory consolidation and increasing synaptic plasticity (Xu et al., 2024). Furthermore, aerobic exercise promotes a beneficial phenotypic shift in hippocampal microglia, influencing both their morphology and proliferation. This regulation not only dampens neuroinflammation but also reinvigorates autophagic pathways. Evidenced by studies in NSE/*htau23* mice, exercise increased Beclin-1 and LC3 expression and decreased p62 accumulation (Kang and Cho, 2015). This rescue of autophagic flux facilitated LTP induction in the CA1 subfield, thereby restoring hippocampal synaptic plasticity.

### 5.2 Exercise-mediated activation of Nrf2 signaling: coupling redox homeostasis with autophagy induction

The Nuclear factor erythroid 2-related factor 2(Nrf2)/antioxidant response element (ARE) signaling pathway constitutes a canonical defense mechanism against oxidative stress, capable of mounting robust cellular antioxidant responses and mitigating neuronal damage. Specifically, ARE—located in the 5′-promoter regions of genes governing antioxidant defense and autophagy—serves as the docking site to trigger these adaptive responses. Nrf2 acts as the master transcriptional regulator orchestrating this protective program. Notably, aerobic exercise has been demonstrated to activate the Nrf2-Keap1 (Kelch-like ECH-associated protein 1) signaling axis, thereby bolstering cellular defense mechanisms (Wang S. et al., 2024; Wang T. et al., 2024). The duration of exercise intervention is a key variable in modulating its beneficial effects against oxidative stress. Generally, short-term exercise may temporarily increase ROS production, whereas long-term regular exercise induces adaptive responses in the body (Ionescu-Tucker and Cotman, 2021). Basally, Nrf2 is kept at low levels through Keap1-mediated ubiquitination. Oxidative stress inhibits this degradation, allowing Nrf2 to stabilize and translocate to the nucleus. There, it partners with Maf proteins to bind the ARE, activating a battery of Phase II antioxidant and repair genes. These include Heme Oxygenase-1 (HO-1), Superoxide Dismutases (SOD1, SOD2), and Glutathione Peroxidases (GPX). Beyond neutralizing free radicals, this pathway also activates DNA repair enzymes such as apurinic/apyrimidinic endonuclease 1 (APE1) via the CREB signaling axis, thereby enhancing cellular DNA repair capacity and shielding neurons from oxidative damage (Yang et al., 2014). Preclinical studies in various rodent models substantiate these mechanisms. In *APP/PS1* mouse models, 12 weeks of aerobic treadmill exercise significantly reduced ROS and Keap1 burden in the hippocampus, which was positively correlated with increased Nrf2 and HO-1 expression (Wang S. et al., 2024; Wang T. et al., 2024). Similar neuroprotective effects have also been observed in aging models. In 15-week-old Wistar rats, moderate-intensity treadmill training significantly reduced ROS levels in hippocampal tissue. This was accompanied by increased expression of key molecules involved in mitochondrial biogenesis and antioxidant defense, including superoxide dismutases (SOD1 and SOD2) and glutathione peroxidases (GPXs) (Marosi et al., 2012). Furthermore, chemically induced AD models exhibit comparable benefits. A 4-week aerobic intervention in Streptozotocin (STZ)-induced rats successfully reduced lipid peroxidation and DNA oxidative damage in the hippocampal CA1 region (Lu et al., 2017). Notably, Nrf2 activation establishes a positive feedback loop with the autophagic machinery through the p62-Keap1 nexus. By upregulating the transcription of autophagy genes (e.g., Atg5, LC3, p62), Nrf2 enhances the clearance of neurotoxic aggregates (Frias et al., 2020; Zhang W. et al., 2021; Zhang X. X. et al., 2021). Synthesizing these findings, aerobic exercise likely leverages this Nrf2/ARE-autophagy axis to mitigate oxidative injury and rescue neuroplasticity.

### 5.3 Exercise activates the SIRT1-FOXO1/3 axis to promote PINK1/Parkin-mediated mitophagy

Silent information regulator 1 (SIRT1) is a class III histone deacetylase that plays a fundamental role in neuroprotection. Research indicates that SIRT1 deacetylates a broad spectrum of transcription factors and coactivators—most notably Forkhead box O (FOXO) proteins and Peroxisome proliferator-activated receptor *γ* coactivator 1-alpha (PGC1-*α*). Through the deacetylation of these autophagy-related targets, SIRT1 induces mitophagy, thereby exerting protective effects on neurons (Eshraghi et al., 2021). The function of SIRT1 is intrinsically linked to nicotinamide adenine dinucleotide (NAD), a coenzyme that oscillates between its NAD + and NADH forms. Consequently, intracellular NAD + levels are critical for balancing mitochondrial clearance with renewal, directly influencing neuronal viability. Specifically, 8 weeks of treadmill running boosted cortical NAD + levels and nuclear SIRT1 expression. This suggests that the exercise-mediated phosphorylation of AMPK restores the NAD+/NADH balance, which in turn acts as a potent stimulus to activate SIRT1 signaling (Huang et al., 2019). The SIRT1–FOXO1/3 axis is well-established for its role in extending cellular longevity and regulating mitochondrial homeostasis (Lim et al., 2017). In parallel, the interaction between PINK1 and Parkin governs the canonical pathway for mitophagy initiation. Specifically, Parkin that ubiquitinates proteins on the OMM. This process effectively targets damaged mitochondria for recognition and subsequent clearance by the autophagic machinery (Cadete et al., 2019). Aerobic exercise improves mitochondrial function in AD animal models. It also exerts neuroprotective effects. These findings are supported by multiple experimental studies. Exercise-induced activation of the SIRT1 axis is further supported by evidence from human muscle-brain axis studies. Khanthong et al. (2024) demonstrated that exercise-induced activation of the SIRT1 axis promotes irisin release after 12 weeks of aerobic exercise, with elevated levels significantly correlated with improved cognitive scores and reduced amyloid burden in AD patients. Using *APP/PS1* transgenic mice as an example, Cui et al. (2023) observed through a 12-week aerobic treadmill training regimen significantly enhances mitochondrial autophagy. This intervention accelerates Aβ clearance and effectively mitigates declines in learning and memory functions (Cui et al., 2023). At the molecular level, Zhao et al. (2023) further revealed that the same exercise regimen activates the SIRT1-FOXO1/3 signaling pathway, thereby promoting the transcriptional expression of PINK1 and Parkin proteins. Activation of this pathway not only optimizes mitochondrial quality control systems but also combines with the upregulation of synapse-related protein expression. Together, these effects lead to reduced amyloid plaque burden in the brain and significant improvements in cognitive behavioral deficits (Cui et al., 2023; Zhao et al., 2023). In summary, current evidence suggests that exercise exerts neuroprotective effects by upregulating the SIRT1-FOXO1/3-PINK1/Parkin axis. Through this mechanism, exercise restores mitochondrial function and reinvigorates mitophagy activity.

### 5.4 Exercise enhances autophagic-lysosomal function via the AdipoR1/AMPK/TFEB signaling axis

Since the execution of autophagy ultimately relies on the fusion with lysosomes to degrade substrates, lysosomal integrity is indispensable for the entire pathway (Huang et al., 2019). Functioning as a central molecular switch, TFEB governs lysosomal biogenesis. Following its activation and nuclear translocation, TFEB targets coordinated lysosomal expression and regulation (CLEAR) elements within gene promoters to drive a comprehensive transcriptional program. This regulatory mechanism not only markedly upregulates lysosome-associated gene expression but also optimizes the overall functional capacity of the lysosomal system (Settembre et al., 2011). Empirical studies indicate that TFEB overexpression upregulates Cathepsin D in the brain tissue of *APP/PS1* mice, effectively restoring lysosomal acidification and enhancing LC3 expression. These molecular changes facilitate the critical fusion between autophagosomes and lysosomes. Conversely, TFEB knockout compromises lysosomal activity, leading to the pathological accumulation of Aβ and Tau aggregates (Song et al., 2020). Specifically, Wang et al. (2022) showed that 5 months of treadmill training induces TFEB nuclear translocation in AD mice. By strengthening the AMPK-dependent interaction between TFEB and Acetyl-CoA synthetase, exercise boosts Cathepsin D/L levels and improves lysosomal competency (Wang et al., 2022). Adiponectin (APN) is a pivotal adipokine secreted by adipose tissue. It possesses the unique ability to permeate the blood–brain barrier (BBB) and bind to its specific receptors, adiponectin receptor 1(AdipoR1) and adiponectin receptor 2 (AdipoR2). Through this receptor interaction, APN exerts modulatory effects on hippocampal neurogenesis and synaptic plasticity (Liu et al., 2019). Demonstrating the critical role of this axis, Jian et al. reported that a 12-week systemic treadmill exercise regimen orchestrates autophagy-lysosomal homeostasis through AdipoR1/AMPK/TFEB signaling. This modulation resulted in enhanced neuronal plasticity in AD mice. Key molecular evidence included elevated Beclin1, enhanced LC3-II conversion, and decreased p62 burden, which collectively supported the observed remodeling of dendritic spines (Jian et al., 2022).

### 5.5 Integrative regulation: the crosstalk of autophagy signaling networks in exercise

**Figure 1:**
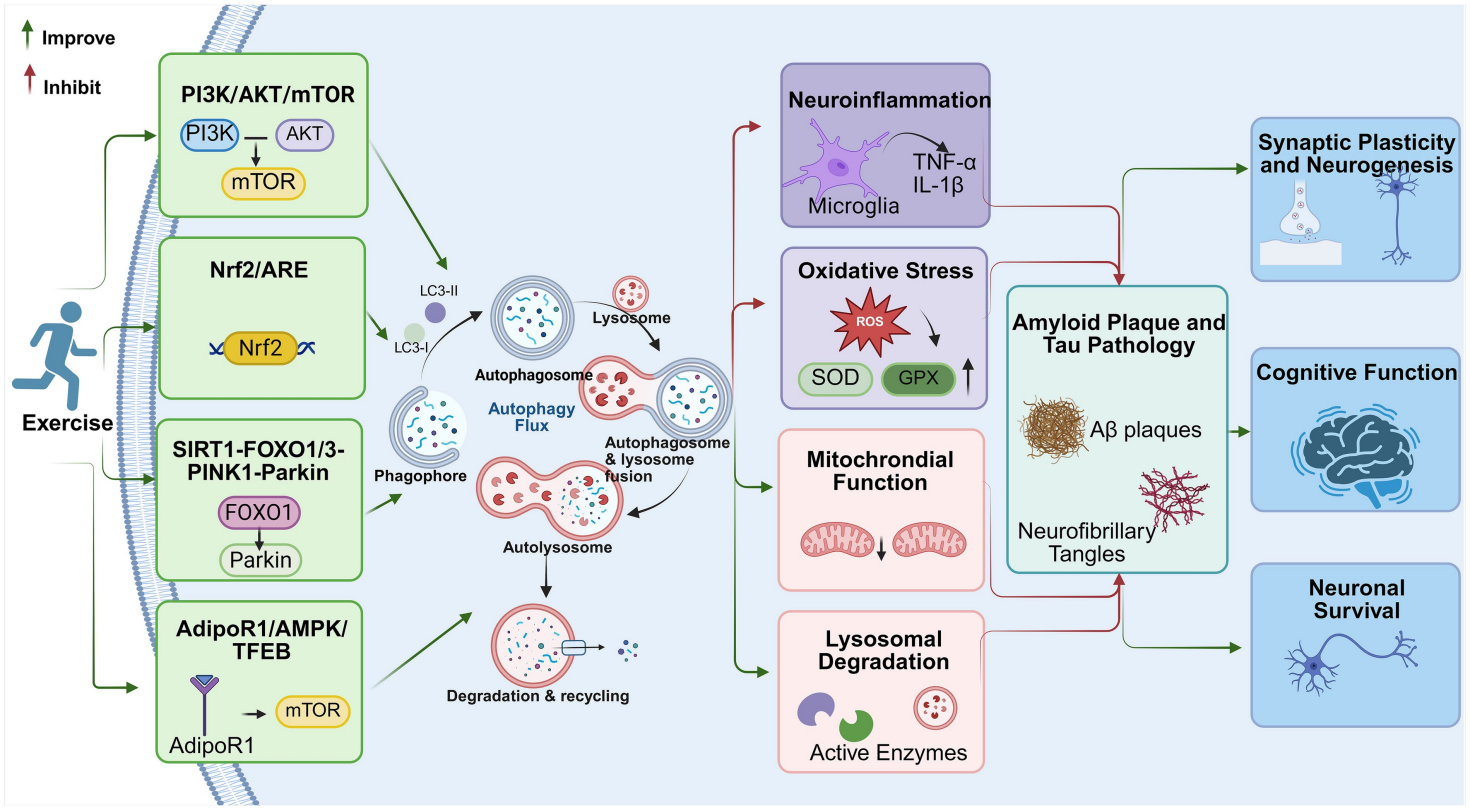
Integrated signaling mechanisms linking exercise to autophagy and neuroplasticity in Alzheimer’s disease. This schematic illustrates the multi-target regulatory network triggered by aerobic exercise within the neuronal environment: exercise intervention synergistically activates four core signaling axes, including the PI3K/AKT/mTOR pathway, the Nrf2/ARE antioxidant defense pathway, the SIRT1-FOXO1/3-PINK1-Parkin mitochondrial autophagy signaling pathway, and the AdipoR1/AMPK/TFEB pathway. These cascading signals synergistically restore the autophagic flux by promoting LC3-II lipidation and efficient autophagosome-lysosome fusion. Enhanced degradation capacity significantly reduces neuroinflammatory cytokines (TNF-α, IL-1β) and ROS levels while improving mitochondrial function and lysosomal enzyme activity. Ultimately, the effective clearance of neurotoxic β-amyloid (Aβ) plaques and neurofibrillary tangles (p-Tau) promotes synaptic remodeling and neurogenesis, thereby alleviating cognitive decline and improving neuronal survival in AD. Created in BioRender. Qian, L. (2026).

While the preceding sections have delineated the individual contributions of specific mediators, our discussion is limited to certain signaling pathways within a complex, integrated regulatory network (Figure 1). Within this intricate regulatory network, primarily characterized in AD rodent models, AMPK functions as the metabolic “master switch” that serves as a central hub coordinating exercise-induced autophagy. Specifically, aerobic exercise-activated AMPK suppresses mTOR activity, thereby relieving the inhibition on autophagy initiation (Mary et al., 2025). Furthermore, the autophagy receptor p62 serves as a pivotal molecular bridge connecting the autophagic machinery with the antioxidant defense system. By activating the “p62-Keap1-Nrf2” axis, p62 triggers Nrf2-mediated antioxidant responses, effectively disrupting the vicious cycle between oxidative stress and pathological protein aggregation (Jiang and Mizushima, 2015). Simultaneously, AMPK synergizes with the NAD+/SIRT1 axis to cooperatively activate downstream FOXO1/3. This signaling cascade drives the PINK1/Parkin pathway, thereby maintaining synaptic energy supply. Furthermore, during the late stage of autophagy, AMPK extends its regulation by promoting the nuclear translocation of TFEB, which enhances lysosomal biogenesis and degradative capacity (Settembre et al., 2011). In conclusion, by orchestrating the remodeling of this multidimensional signaling web, exercise systematically re-establishes neuronal homeostasis and promotes robust neural plasticity.

## 6 Summary and perspectives

Given that autophagic homeostasis is central to the regulation of neuronal plasticity in AD pathology, targeting this machinery constitutes a compelling therapeutic strategy. Synthesizing these findings, exercise appears to act as a potent modulator that boosts autophagic flux and improves neural plasticity, ultimately mitigating cognitive decline. By recalibrating the signaling networks discussed above, exercise effectively dampens neuroinflammation, reduces oxidative stress, and revitalizes mitochondrial and lysosomal dynamics. However, the regulatory architecture of autophagy remains highly complex. Future studies are thus required to dissect the specific molecular interactions through which exercise exerts its therapeutic effects in AD. However, the benefits of exercise are limited by intervention timing and modality. Early implementation is paramount, as efficacy diminishes in advanced AD pathology. Future investigations should therefore focus on defining the optimal timing and types of physical activity that best modulate autophagy and neuroplasticity. This will help establish precision exercise prescriptions for preserving cognitive function in Alzheimer’s patients.
